# Assessment of a manual therapy and acupressure method as a treatment of nonspecific low back pain: A prospective, observational and non-interventional cohort study

**DOI:** 10.1097/MD.0000000000040891

**Published:** 2024-12-20

**Authors:** Gilles Ducret, Marc Guillaume, Yann Fardini, Sandrine Vejux, Hassène Chaabi

**Affiliations:** a Association of Biokinergie® Practitioners, Saint-Lunaire, France; b Soladis Clinical Studies, Roubaix, France; c Osteopathic Clinic, Douarnenez, France; d Physiotherapy Clinic, Antibes, France.

**Keywords:** acupressure, Biokinergie, integrative and systemic method, Low back pain, manual therapy, pain

## Abstract

The first-line treatment for nonspecific low back pain (LBP) relief is physical exercise; however, there is no uniformity in recommendations regarding the type of exercise, and physicians predominantly prescribe pharmacological treatments. This creates a treatment gap in non-pharmacological management of LBP. Preliminary data suggest that manual therapy and acupressure could be relevant therapeutic options. The primary objective of this study was to describe the evolution of pain in patients with nonspecific LBP persisting for at least 4 weeks who received 2 treatment sessions combining manual therapy with acupressure (Biokinergie® method) as part of their routine management. In this prospective, observational, and non-interventional study, participants were monitored on the days of manual therapy session delivery (initial visit on Day 0 and follow-up visit on Day 21). Follow-up assessments occurred 3 days and 3 weeks post-therapy sessions. A total of 114 participants suffering from LBP for a median duration of 11.9 weeks were enrolled from May 2021 to May 2023. The intensity of average pain experienced over a 24-hour period was significantly reduced on Day 24 (−36.0 ± 27.2 mm on a visual analog scale of 100 mm, *P* < .001), with 82 (75.2%) participants reporting a decrease of at least 20 mm. Participants reported significant reductions in pain (−25.9 ± 23.9 mm on Day 3, −29.7 ± 26.7 mm on Day 21, and −40.9 ± 28.3 mm on Day 42, *P* < .001) and functional disability (Roland-Morris Disability Questionnaire score: −5.4 ± 0.4 points on Day 21 and −7.4 ± 0.4 points on Day 42, *P* < .001). This was associated with an improved Dallas Pain Questionnaire score, indicating a reduced impact of LBP on daily activities (−27.2 ± 2.0% on Day 21 and −35.8 ± 2.0% on Day 42, *P* < .001). Posttreatment, most participants (86.3%) reported reduced analgesic intake compared to baseline, and 83.3% were very satisfied with the therapy. A significant and clinically relevant reduction in lumbar pain was observed after 2 sessions of manual therapy combined with acupressure, paving the way for future clinical research.

## 1. Introduction

Low back pain (LBP) is a common health issue,^[1]^ classified as nonspecific LBP when lumbar pain cannot be attributed to a specific underlying condition.^[2]^ LBP can become a barrier to employment, as it is the second most common reason for consultation with general practitioners in France and accounts for 30% of work stoppages lasting more than 6 months related to spinal problems.^[3]^ In Western countries, societal costs for LBP are estimated to be 1% to 2% of the gross national product, with 80% to 90% of these costs attributed to productivity loss and employee disability.^[4]^ The French National Authority for Health reports that 90% of patients recover from LBP within 4 to 6 weeks,^[3]^ either spontaneously or with physical activity. Therefore, the major socioeconomic burden of LBP comes from the 10% of patients who experience persistent pain that does not resolve within this 6-week period. LBP can be categorized based on its duration: acute LBP, lasting 4 weeks or less; subacute LBP, persisting between 4 and 12 weeks; and chronic LBP, exceeding 3 months. In France, the first-line treatment for nonspecific LBP relief is physical exercise^[5–8]^; however, there is no uniformity in the recommended exercise type.^[9]^ Additionally, physicians predominantly prescribe pharmacological treatments.^[10]^ A significant unmet need persists for non-pharmacological treatment options that effectively reduce pain and improve functional disability,^[11]^ while limiting the environmental burden of analgesics.^[12]^

Recent studies have highlighted that manual therapy^[13–15]^ and acupressure^[16–18]^ could be considered treatment options for LBP. Among these approaches, Biokinergie® is an integrative manual therapy offering a noninvasive and non-instrumental approach. This method synthesizes the therapeutic goals of osteopathy, manual therapy, massage therapy, and traditional Chinese medicine. It is characterized by its gentle mobilizations and manual stimulation of tissues at strategic points, referred to as points of hypertonia. Easily identifiable through palpation, these points indicate areas of tension in the tissues and exhibit a slight spiral deformation in response to pressure. Points of hypertonia may be found in different body regions, including Chapman’s points,^[19]^ as well as trigger points, which are reflex points used in physiotherapy and osteopathy to release muscle contractures and myofascial pain.^[20,21]^ Points of hypertonia correspond to muscles and ligaments whose tensions can cause somatic dysfunctions^[22]^ that could maintain pain and restrict mobility. They can also be identified at acupuncture points, which are sites for stimulating afferent nerve fibers capable of producing an analgesic effect.^[16,23,24]^ As a global approach, the therapeutic gesture in Biokinergie® focuses on the articular, myofascial, and traditional Chinese medicine energetic systems, which are guided by the presence of points of hypertonia. This therapy targets 3 primary objectives: the release of mobility restrictions, the alleviation of myofascial disorders, and the manual (needle-free) stimulation of acupuncture points indicated for the treatment of pain, inflammation, visceral dysfunctions, and biological disorders. Pressure applied to these tension points, respecting the tissue’s natural deformability, initiates corrective spiral mechanisms tailored to address the dysfunctions being treated. These pressures aim to stimulate dysfunctional neuroreceptors in the somatosensory system, triggering the inhibition of contracted muscles that cause structural misalignments and mobility restrictions. The subsequent correction involves gentle mobilizations to release the dysfunction, in alignment with the osteopathic principle of facilitating tissues toward a balanced still point. Originating in France in the 1980s through the *Centre d’Enseignement et de Recherche en Biokinergie®*, the method has since been adopted by approximately 2000 health professionals and osteopaths. These practitioners have undergone a comprehensive 580-hour training program, culminating in a certification that validates their theoretical knowledge in neuro-musculo-aponeurotic anatomy and physiology, the meridian system (including primary and secondary meridians, as well as the major meridians) of Chinese medicine, and their practical skills. This certification process is assessed by a jury of professionals highly experienced in Biokinergie®, manual therapy, osteopathy, and Chinese massage.

A pilot study involving athletes who received 1 session of this combination of manual therapy and acupressure suggested that this method can influence metabolic adaptation and the cardiorespiratory system.^[25]^ To date, there is no data on the use of this method for the pain management of patients with nonspecific LBP, despite them representing a notable part of the practice population. Gathering real-life data could provide a basis for future large-scale experimental evaluation. To this end, we conducted the first prospective, observational, and non-interventional study reporting the use of this manual therapy associated with acupressure (Biokinergie® method) in the management of LBP. The primary objective was to describe the evolution of lumbar pain in patients with nonspecific LBP persisting for at least 4 weeks who underwent a 2-session therapeutic program combining manual therapy with acupressure.

## 2. Methods

### 2.1. Ethics

The study was conducted in compliance with legal and regulatory requirements. The protocol received approval on February 8, 2021, from a French Independent Ethics Committee (*Comité de Protection des Personnes Est-II*). This observational, non-interventional study adhered to the Research Standard MR-003, “Research in the Field of Health Without Collection of Consent,” established by the French Data Protection Authority (*Commission Nationale de l’Informatique et des Libertés*–CNIL), in accordance with the French law of January 6, 1978, as modified by the General Data Protection Regulation (EU) 2016/679. Participants were informed about the research and understood that participation was voluntary. They signed a non-opposition form, as formal written informed consent was not required.

### 2.2. Study design

This was a multicenter, prospective, observational, and non-interventional study conducted in France with 29 healthcare professionals from May 7, 2021, to May 10, 2023. Participants were followed during their routine care over 2 visits, coinciding with therapy sessions: the initial visit on Day 0 (inclusion visit and “Session 1”) and a follow-up visit 3 weeks later on Day 21 (Day 21 ± 3 days, “Session 2”). Follow-up questionnaires were completed 3 days post-session (on Days 3 and 24) and 3 weeks post-session (on Days 21 and 42) after each therapy session.

### 2.3. Participants

Eligible participants were adults with nonspecific LBP persisting for at least 4 weeks and with a pain intensity of at least 40 mm on a 100 mm visual analog scale (VAS). They were scheduled for a 2-session program combining manual therapy with acupressure (Biokinergie® method), independent of their study participation.

Exclusion criteria included lumbar pain related to an identified pathology (e.g., spondylarthritis, significant scoliosis > 50° Cobb, grade I or II spondylolisthesis, dysmorphogenesis, transitional anomalies, vertebral hyperostosis, or neoplasia), history of lumbar surgery, or pregnancy.

### 2.4. Two-session therapy program for LBP treatment

The therapy sessions, administered by a physiotherapist or osteopath, were scheduled 3 weeks apart, with Session 1 on Day 0 and Session 2 on Day 21 ± 3 days. This therapeutic program was determined based on participants’ symptoms prior to inclusion, independent of study participation. Given the observational and non-interventional nature of the study, the conduct of therapy sessions was left to the practitioner’s discretion, following their routine practice.

Typically, each session began with palpation assessments and mobility tests of areas with joint mobility restrictions and muscular tensions. These areas, influenced by gravity, tension propagation within muscular chains, and neuro-muscular dysfunctions, were associated with disorders in the affected zone. Assessments included anamnesis, a postural evaluation of the standing patient, and both active and passive mobility tests, as well as the identification of myofascial hypertonia points and reactive acupuncture points (points that respond to manual pressure with tissue tension).

The practitioner used gentle mobilizations and manual stimulation at the identified hypertonia points, which responded to pressure with a slight spiral deformation of the tissues. Acupuncture points were stimulated, and joint and myofascial tensions were simultaneously addressed by guiding the structure back to facilitated positional release^[26–28]^ following muscle relaxation through light corrective mobilizations. As in the initial assessment, tests were repeated at the session’s end to verify restoration of overall body balance, joint mobility, and tissue flexibility in the dysfunctional areas, particularly focusing on the lumbar region. The typical session duration was approximately 60 minutes.

### 2.5. Study outcomes

The primary outcome was the mean change from baseline (Day 0) to 3 days after completing the 2-session therapy program (Day 24) in the average intensity of LBP experienced over the past 24 hours.

Secondary outcomes included the mean change in the average intensity of LBP over a day, from baseline (Day 0) to each follow-up visit (Days 3, 21, 24, and 42). Similarly, the mean changes in average LBP intensity over a week and in the worst LBP intensity over a week were assessed. We also examined the mean change in functional disability level and the mean change in the percentage impact of LBP on participants’ quality of life (QoL). Additionally, the mean change in analgesic consumption frequency from baseline was evaluated. Furthermore, participant satisfaction with the therapy and the relationship between therapeutic expectations and outcomes were assessed.

The evolution of LBP, functional disability, and the impact of LBP on participants’ QoL were analyzed according to the risk of LBP chronicity.

### 2.6. Data sources/measurements

Participants recorded their lumbar pain intensity throughout the study using a 100 mm VAS: at baseline (Day 0, before “Session 1”), and then 3 days and 3 weeks after each therapy session (on Day 3 and Day 24 ± 3 days for “Session 1,” and on Day 21 ± 3 days and Day 42 ± 3 days for “Session 2”).

Functional disability was assessed using the French version of the Roland-Morris 24-item Disability Questionnaire (RMQ),^[29]^ validated for chronic LBP follow-up.^[30]^ The RMQ score ranges from 0 point (no disability) to 24 points (maximum disability). A mean change in the RMQ score, equal or >2 points, was considered clinically relevant.^[31]^

The percentage impact of LBP on participants’ QoL was measured using the Dallas Pain Questionnaire (DPQ), which is validated for chronic LBP follow-up.^[32]^ This questionnaire evaluates the impact of pain in percentage terms (0 to 100%) across daily activities, work-leisure activities, anxiety-depression, and social life.

Functional disability and the impact of lumbar pain on participants’ QoL were documented at baseline (Day 0) and on Day 21 ± 3 days and Day 42 ± 3 days.

The risk of developing chronic LBP was assessed at baseline (Day 0) using the STarT back screening tool (SBST), with a global score ranging from 0 to 9 points, where the risk of chronicity increases with the score,^[33]^ and categorized as “low,” “intermediate,” or “high.”

The mean changes from baseline (week prior to study inclusion) in analgesic consumption frequency were reported using a 5-level Likert scale: “much more,” “a little more,” “the same,” “a little less,” and “much less.” Analgesic consumption was documented for the week preceding study inclusion (before Day 0) and on Day 21 ± 3 days and Day 42 ± 3 days.

Participant satisfaction with therapy delivery and therapeutic outcome expectations were reported using a 5-level Likert scale: “very dissatisfied,” “dissatisfied,” “neutral,” “satisfied,” and “very satisfied.” Satisfaction with therapy delivery was collected at baseline (Day 0) and on Day 21 ± 3 days and Day 42 ± 3 days, while therapeutic outcome expectations were documented on Day 42 ± 3 days.

### 2.7. Bias

Inclusion and exclusion criteria were established to develop a cohort with homogeneous characteristics, and eligible participants were included consecutively. Clinical and sociodemographic variables were collected at baseline to describe the cohort, including individual risk of LBP chronicity. Validated questionnaires were employed to assess pain and its impact on participants’ daily lives. For statistical analysis, changes from baseline were modeled using a mixed model for repeated measures (MMRM), with time as a fixed factor and baseline value as a covariate to account for baseline variation.

### 2.8. Study size

The main objective of this study was to describe the evolution of lumbar pain after a 2-session therapy program. A recent study demonstrated a mean reduction of 33 mm in pain within a few weeks of manual therapy for patients with chronic LBP.^[34]^ Similarly, a randomized controlled trial showed a mean decrease of 28.7 ± 30.3 mm in lumbar pain after 8 weeks of acupuncture treatment in chronic LBP (compared to 6.9 ± 22.0 mm in the control group).^[35]^ A lumbar pain reduction exceeding 20 mm is considered clinically relevant.^[36,37]^ Based on an expected standard deviation (SD) of 30 mm, a sample size of 99 patients was necessary to estimate an average reduction of 20 mm with a 95% confidence interval (95% CI) ranging from 14 to 26 mm. Accounting for a 15% loss to follow-up, 114 participants were recruited.

### 2.9. Statistical methods

Statistical analyses were conducted on the full-analysis set (FAS) population, comprising all included participants who received at least 1 therapy session.

Quantitative variables were described using the mean with standard error of the mean (SEM) or SD, median (med) with quartiles (Q1 and Q3). Qualitative variables were described by frequency. Missing data were not replaced.

The primary outcome was expressed by the mean difference in LBP intensity over the last 24-hour period at Day 24 compared to baseline. A paired Student *t* test was used to compare the mean values at Day 24 with those at baseline; a *P*-value was <.05 was considered statistically significant. As a sensitivity analysis, the change from baseline in average LBP intensity over a day was modeled using a MMRM, with baseline value as a covariate and time (Days 3, 21, 24, and 42) as fixed factors to account for variability across measurement times.

Several covariance structures were tested: compound symmetry (CS); first-order autoregressive (AR(1): equal variances and exponentially decreasing correlations); Toeplitz (TOEP: equal variances with a separate correlation for each level of separation between time points); variance-covariance (VC); and unstructured (UN). The model with the lowest Akaike Information Criterion (AIC) value was selected as the preferred model. Normality of Cholesky residuals was verified using a Shapiro–Wilk test for fewer than 50 values or a Kolmogorov-Smirnov test otherwise. Normality was rejected at an alpha risk level of 1%, with a graphical check of scaled residuals conducted. If normality was rejected, a Bootstrap confidence interval was applied.

Secondary outcomes, including daily and weekly LBP intensity, worst weekly LBP intensity, functional disability, and QoL impact, were analyzed using MMRM.

The proportion of participants achieving a clinically significant pain reduction and those reporting decreased analgesic use were presented with 2-sided 95% CIs (Clopper-Pearson method). An exploratory analysis based on individual risk of LBP chronicity was also conducted to assess changes in pain, functional disability, and QoL, using the same analytical approach as the primary analysis.

All statistical analyses were conducted with SAS 9.4 software (SAS Institute, Cary, NC, USA).

## 3. Results

### 3.1. Participants

**Figure 1.**
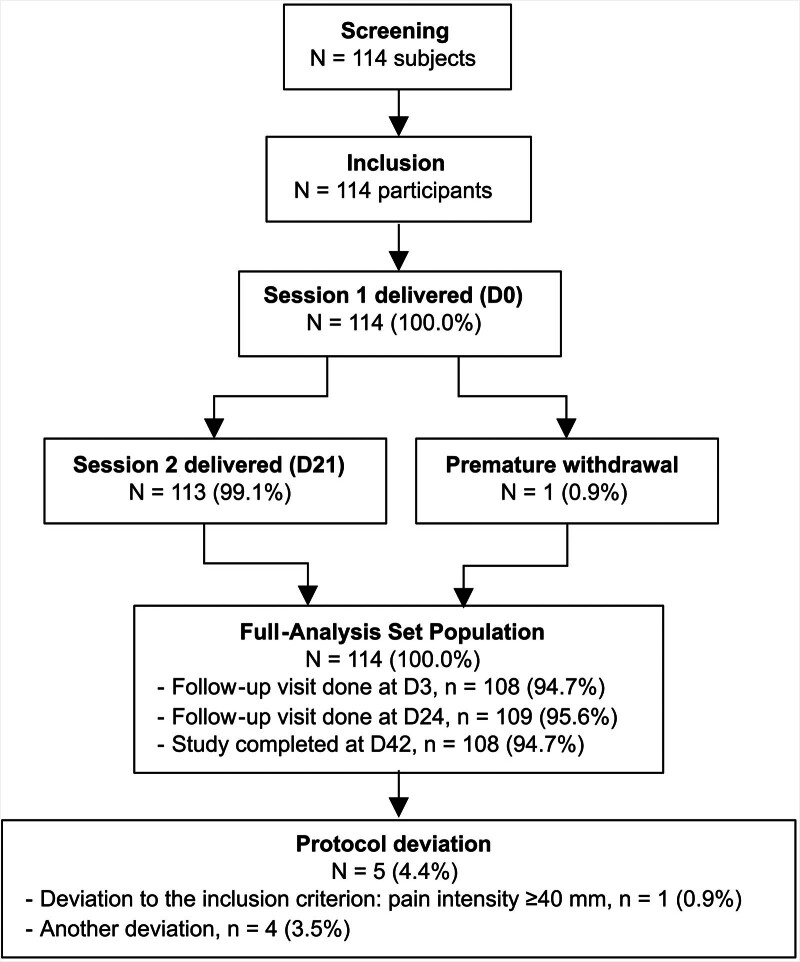
Participants flow chart. D0 = Day 0, study inclusion visit; D3 = Day 3, 3 d after the first treatment (session 1) delivery; D21 = Day 21, 3 wk after the first treatment (session 1) delivery; D24 = Day 24, 3 d after the second treatment (session 2) delivery; D42 = Day 42, 3 wk after the second treatment (session 2) delivery; n = sample size; N = total number of participants.

One hundred and fourteen participants were screened and included in the study (from May 7, 2021, to May 10, 2023). The participant flow chart is summarized in Figure 1.

Session 1 (on Day 0) was delivered to all participants (n = 114, 100.0%). Session 2 (on Day 21) was delivered to 113 participants (99.1%). One participant (0.9%) prematurely withdrew from the study due to a diagnosis of spondylolisthesis and did not complete the visit for Session 2.

Follow-up questionnaires to be answered after Session 1 (on Day 3), after Session 2 (on Day 24), and at the study’s end (on Day 42) were completed by 94.7% (n = 108), 95.6% (n = 109), and 94.7% (n = 108) of the participants, respectively.

Results were obtained from statistical analyses conducted on the FAS population (N = 114).

### 3.2. Characteristics of study participants

Population characteristics at baseline are described in Table 1. Most participants were female (n = 79, 69.3%), with a mean age (± SD) of 50.8 ± 15.6 years and a normal body mass index of 24.8 ± 4.4 kg/m^2^. Relevant medical histories included traumatic episodes (n = 44, 39.6%), sports injuries (n = 31, 27.7%), and osteoporosis (n = 8, 7.1%).

**Table 1:** Demographics and baseline characteristics of the population.

Variable	Full-analysis set population (N = 114)
Demographics, N (missing)	114 (0)
Age, yr	50.8 ± 15.6
Female	79 (69.3%)
BMI, kg/m^2^	24.8 ± 4.34
Medical history	
Traumatic episodes (work accident, car accident, etc.)	44 (39.6%)
Sports injuries	31 (27.7%)
Osteoporosis	8 (7.1%)
History of LBP, N (missing)	109 (5)
Duration of LBP, wk	11.9 [6.7; 113.9]
Risk factor for LBP chronicity, N (missing)	101 (0)
Stress	61 (60.4%)
Anxiety	53 (52.5%)
Prolonged postures	49 (48.5%)
Carrying heavy loads	40 (39.6%)
Being overweight or obese	20 (19.8%)
Another factor	14 (13.9%)
LBP characteristics	
Mechanical type (daytime pain increasing with physical activity)	82 (73.2%)
Inflammatory type (nighttime pain leading to early morning wake-up)	61 (53.5%)
Pain staring at the lumbosacral junction	103 (91.2%)
Presence of radiating pain	80 (70.8%)
LBP intensity assessed by a VAS of 100 mm, N (missing)	114 (0)
Pain experienced over a d	61.2 ± 17.0
Pain experienced over a wk	60.5 ± 15.5
Worst pain experienced over a wk	74.6 ± 15.9
Risk of LBP chronicity according to SBST score, N (missing)	112 (2)
Low	20 (17.9%)
Intermediate	38 (33.9%)
High	54 (48.2%)
Analgesic intake, N (missing)	114 (0)
At least 1 drug taken during the last wk	69 (60.5%)
At least 1 non-opioid drug taken per d	49 (43.0%)
At least 1 mild opioid drug taken per d	13 (11.4%)
At least 1 strong opioid drug taken per d	1 (0.9%)
Level of functional disability assessed by 24-item RMQ, N (missing)	114 (0)
Score in points	11.0 [6.0; 14.0]
Impact percentage of LBP on participants’ QoL assessed by DPQ, N (missing)	114 (0)
Daily activities	55.6% ± 19.1%
Work-leisure activities	51.1% ± 27.2%
Anxiety-depression	33.5% ± 26.2%
Social life	20.4% ± 21.7%

The values represent N (missing), n (%), mean ± SD, or median [Q1; Q3].

The low back pain (LBP) intensity was assessed by a visual analog scale (VAS) of 100 mm.

The risk of developing LBP chronicity was assessed by STarT back screening tool (SBST).

The level of functional disability was assessed by the 24-item Roland Morris Disability Questionnaire (RMQ).

The impact percentage of LBP on the participants’ quality of life (QoL) was assessed by the Dallas Pain Questionnaire (DPQ).

Abbreviations: BMI = body mass index; DPQ = Dallas Pain Questionnaire; LBP = low back pain; N = total number of participants; QoL = quality of life; RMQ = 24-item Roland Morris Disability Questionnaire; SBST = STarT back screening tool; VAS = visual analog scale.

The median duration of LBP at study inclusion was 11.9 weeks. Participants presented several risk factors for chronic LBP, including stress (n = 61, 60.4%), anxiety (n = 53, 52.5%), prolonged postures (n = 49, 48.5%), carrying heavy loads (n = 40, 39.6%), and being overweight or obese (n = 20, 19.8%). LBP typically originated at the lumbosacral junction (n = 103, 91.2%) and was predominantly classified as mechanical (n = 82, 73.2%; daytime pain increasing with physical activity) or inflammatory (n = 61, 53.5%; nighttime pain leading to early morning wake-up), with radiating pain in many cases (n = 80, 70.8%). The mean LBP intensity over a week was 60.5 ± 15.5 mm, with the worst LBP reaching 74.6 ± 15.9 mm. Approximately half of the participants (n = 54, 48.2%) had a high risk of LBP chronicity, 1-third (n = 38, 33.9%) had an intermediate risk, and slightly less than one-fifth (n = 20, 17.9%) had a low risk. Most participants (n = 69, 60.5%) reported taking at least 1 analgesic for LBP in the week prior to study inclusion. The mean RMQ score was 10.6 ± 5.23 points, indicating a medium level of functional disability. LBP had a significant impact on participants’ QoL, notably affecting daily activities (55.6 ± 9.1%) and work-leisure activities (51.1 ± 27.2%) as measured by the DPQ.

### 3.3. Primary outcome: significant reduction of LBP 3 days post-therapy

The mean LBP intensity over the last 24 hours was reduced from 61.2 ± 17.0 mm pre-therapy to 24.9 ± 22.5 mm 3 days posttreatment on Day 24, resulting in a statistically significant mean difference from baseline of −36.0 ± 27.2 mm (Student *t* test: *P* < .001). Sensitivity analysis across all timepoints confirmed the significant decrease (*P* < .001): −25.7 mm (95% CI [−29.6, −21.9]) on Day 3, −29.6 mm (95% CI [−33.8, −25.4]) on Day 21, −35.8 mm (95% CI [−40.1, −31.6]) on Day 24, and −41.0 mm (95% CI [−45.2, −36.9]) on Day 42 (Table 3).

### 3.4. Secondary outcomes: reduction in LBP and functional disability

Three days post-therapy, 75.2% (n = 82) of participants reported a reduction in LBP intensity of at least 20 mm, increasing to 78.5% (n = 84) 3 weeks post-therapy (Table 2).

**Table 2:** Evolution of low back pain intensity and functional disability.

Full-analysis set population N = 114	Baseline	Post-session 1		Post-session 2	
	Day 0	Day 3	Day 21	Day 24	Day 42
LBP intensity over a day, N (missing)	114 (0)	109 (5)	112 (2)	109 (5)	107 (7)
Mean ± SD	61.2 ± 17.0	35.0 ± 20.6	31.2 ± 22.4	24.9 ± 22.5	19.7 ± 21.3
Change from baseline (MMRM)					
Mean ± SEM		−25.7 ± 1.9	−29.6 ± 2.1	−35.8 ± 2.1	−41.0 ± 2.1
95% CI		[−29.6; −21.9]	[−33.8; −25.4]	[−40.1; −31.6]	[−45.2; −36.9]
Fixed effect of time		*P* < .001			
Proportion of participants with a reduction of at least 20 mm in LBP, N (missing)			112 (2)	109 (5)	107 (7)
n (%)			74 (66.1%)	82 (75.2%)	84 (78.5%)
LBP intensity over a week, N (missing)	114 (0)		112 (2)		106 (8)
Mean ± SD	60.5 ± 15.5		31.0 ± 20.6		21.7 ± 21.4
Change from baseline (MMRM)					
Mean ± SEM			−29.3 ± 2.0		−38.6 ± 2.0
95% CI			[−33.2; −25.3]		[−45.6; −34.6]
Fixed effect of time		*P* < .001			
Worst LBP intensity over a week, N (missing)	114 (0)		112 (2)		105 (9)
Mean ± SD	74.6 ± 15.9		43.1 ± 26.9		27.2 ± 25.1
Change from baseline (MMRM)					
Mean ± SEM			−31.4 ± 2.4		−47.3 ± 2.5
95% CI			[−36.2; −26.5]		[−52.2; −42.3]
Fixed effect of time		*P* < .001			
Functional disability (RMQ), N (missing)	114 (0)		112 (2)		108 (6)
Mean ± SD	10.6 ± 5.2		5.1 ± 4.7		3.2 ± 3.9
Change from baseline (MMRM)					
Mean ± SEM			−5.4 ± 0.4		−7.4 ± 0.4
95% CI			[−6.2; −4.6]		[−8.1; −6.6]
Fixed effect of time		*P* < .001			

The values represent N (missing), n (%), mean ± SD, mean ± SEM and 95% IC, or *P* value.

The low back pain intensity was assessed by a visual analogic scale of 100 mm.

The level of functional disability was assessed by the 24-item Roland Morris Disability Questionnaire (RMQ).

The differences were calculated between the parameter value at a given timepoint and that at baseline.

Change from baseline was calculated using a mixed model for repeated measures (MMRM) with baseline value as covariate and time as fixed factors.

Proportion expressed with a 95% CI was calculated using the Clopper-Pearson method.

Abbreviations: 95% CI = 95% confidence interval; LBP = low back pain; MMRM = mixed model for repeated measures; N = total number of participants; RMQ = Roland Morris Disability Questionnaire; SD = standard deviation; SEM = standard error of the mean.

Similar mean changes from baseline were observed in weekly LBP intensity (−38.6 mm, 95% CI [−45.6, −34.6] at Day 42) and in the worst weekly LBP intensity (−47.3 mm, 95% CI [−52.2, −42.3] on Day 42) (*P* < .001, Table 2).

A statistically significant reduction in functional disability was also noted over time (*P* < .001, Table 3), with the mean RMQ score decreasing by −5.4 (95% CI [−6.2, −4.6]) points on Day 21 and −7.4 (95% CI [−8.1, −6.6]) points on Day 42 (Table 2).

**Table 3:** Evolution of the impact of low back pain on the participants’ quality of life and evolution of analgesics intake.

Full-analysis set population (N = 114)	Baseline	Post-session 1	Post-session 2
	Day 0	Day 21	Day 42
Impact percentage of LBP on daily activities, N (missing)	114 (0)	112 (2)	107 (7)
Mean ± SD	55.6 ± 19.1	28.0 ± 21.7	19.4 ± 21.6
Change from baseline (MMRM)			
Mean ± SEM		−27.2 ± 2.0	−35.8 ± 2.0
95% CI		[−31.2; −23.2]	[−39.8; −31.8]
Fixed effect of time		*P* < .001	
Impact percentage of LBP on work-leisure activities, N (missing)	114 (0)	112 (2)	106 (8)
Mean ± SD	51.1 ± 27.2	24.2 ± 24.7	18.6 ± 23.2
Change from baseline (MMRM)			
Mean ± SEM		−26.3 ± 2.2	−31.8 ± 2.2
95% CI		[−30.5; −22.0]	[−36.1; −27.4]
Fixed effect of time		*P* = .08	
Impact percentage of LBP on anxiety-depression, N (missing)	114 (0)	112 (2)	106 (8)
Mean ± SD	33.5 ± 26.2	16.0 ± 20.7	10.3 ± 17.3
Change from baseline (MMRM)			
Mean ± SEM		−16.9 ± 1.6	−22.9 ± 1.6
95% CI		[−20.0; −13.7]	[−26.1; −19.6]
Fixed effect of time		*P* < .001	
Impact percentage of LBP on social life, N (missing)	114 (0)	112 (2)	106 (8)
Mean ± SD	20.4 ± 21.7	9.8 ± 14.8	5.8 ± 13.0
Change from baseline (MMRM)			
Mean ± SEM		−9.8 ± 1.2	−14.0 ± 1.2
95% CI		[−12.1; −7.5]	[−16.4; −11.6]
Fixed effect of time		*P* < .001	
Frequency (n, %) of analgesics intake compared to baseline, N (missing)		101 (13)	95 (19)
Much more		0 (0.0%)	0 (0.0%)
A little more		0 (0.0%)	1 (1.1%)
The same		27 (26.7%)	12 (12.6%)
A little less		22 (21.8%)	13 (13.7%)
Much less		52 (51.5%)	69 (72.6%)
Proportion of participants reporting having taken a little less or much less analgesics		74 (73.3%)	82 (86.3%)
95% CI		[63.5%; 81.6%]	[77.7%; 92.5%]

The values represent N (missing), mean ± SD, mean ± SEM, n (%), or 95% IC.

The impact percentage of LBP on the participants’ quality of life (QoL) was assessed by the Dallas Pain Questionnaire (DPQ).

Change from baseline was calculated using a mixed model for repeated measures (MMRM) with baseline value as covariate and time as fixed factors.

Proportion expressed with a 2-sided 95% CI (Clopper-Pearson method).

Abbreviations: 95% CI = 95% confidence interval; DPQ = Dallas Pain Questionnaire; LBP = low back pain; MMRM = mixed model for repeated measures; N = total number of participants; n = sample size; QoL = quality of life; SD = standard deviation; SEM = standard error of the mean.

### 3.5. Secondary outcomes: Improvement in the participants’ quality of life and satisfaction

The impact of LBP on daily activities decreased significantly throughout the study (*P* < .001, Table 3), with a mean change from baseline in impact percentage going from −27.2% (95% CI [−31.2, −23.2]) on Day 21 to −35.8% (95% CI [−39.8, −31.8]) on Day 42. Similarly, the impact on work-leisure activities was reduced by −26.3% (95% CI [−30.5, −22.0]) on Day 21 and −31.8% (95% CI [−36.1, −27.4]) on Day 42, although this was not statistically significant (*P* = .08, Table 3).The impact on anxiety-depression also decreased significantly (*P* < .001, Table 3), from −16.9% (95% CI [−20.0, −13.7]) on Day 21 to −22.9% (95% CI [−26.1, −19.6]) on Day 42. Social life impact also diminished post-therapy (*P* < .001, Table 3), from −9.8% (95% CI [−12.1, −7.5]) on Day 21 to −14.0% (95% CI [−16.4, −11.6]) on Day 42.

Only 1 participant (1.1%) reported increased analgesic use compared to pre-study levels (Table 3). Additionally, 27 participants (26.7%) on Day 21 and 12 participants (12.6%) on Day 42 reported using the same number of analgesics. Most participants reduced analgesic intake following treatment: 73.3% (95% CI [63.5, 81.6]) by Day 21 and 86.3% (95% CI [77.7, 92.5]) by Day 42 (Table 3). After Session 1, 84 participants (77.8%) expressed being very satisfied with the care provided, with satisfaction increasing to 90 participants (83.3%) after Session 2 (Table S1, Supplemental Digital Content, http://links.lww.com/MD/O188). Thirteen participants (12.0%) were somewhat satisfied, 2 (1.9%) were neutral, and 3 (2.8%) were completely dissatisfied. Reasons for dissatisfaction were unavailable. Furthermore, most participants reported being very satisfied (n = 60, 56.6%) or satisfied (n = 29, 27.4%) with their treatment outcome expectations (Table S1, Supplemental Digital Content, http://links.lww.com/MD/O188), while 3 participants (2.8%) were completely disappointed with the therapy outcomes.

### 3.6. Exploratory outcomes: risk of chronicity of LBP and evolution of lumbar pain, functional disability, or quality of life

An exploratory analysis was conducted to examine the relationship between LBP chronicity risk measured at baseline and the evolution of LBP intensity, functional disability, and participants’ QoL. Improvements in each of these parameters were observed, regardless of chronicity risk level at baseline (Table S2, Supplemental Digital Content, http://links.lww.com/MD/O188).

## 4. Discussion

### 4.1. Key results

This observational and non-interventional study reported favorable outcomes for 114 participants with nonspecific LBP who received 2 sessions of combined manual therapy and acupressure. Mean pain levels were significantly and consistently reduced across all time points, and a vast majority of participants reported a pain reduction of at least 20 mm on the VAS. Functional disability, assessed by the RMQ score, showed significant improvement by Day 42. Quality of life also improved, with significant decreases in the impact of LBP on daily activities and anxiety-depression. Most participants reduced their analgesic intake, and high satisfaction rates were recorded post-therapy. To our knowledge, this is the first clinical study investigating the potentially beneficial impact of the Biokinergie® method, which combines manual therapy with acupressure, in the management of nonspecific LBP.

### 4.2. Limitations

This prospective study has limitations due to its observational and non-interventional design, including the lack of a comparison with an active treatment or a control condition. Therefore, it is not possible to rule out nonspecific effects such as the Hawthorne effect, the placebo effect, or regression toward the mean.

A major limitation of the pre-post analysis performed is that the interpretation of results is based solely on the temporal relationship between the outcomes and the intervention. Other variables may have influenced the outcomes beyond the manual therapy.

Many outcomes were self-reported by participants, which can introduce subjectivity, recall, and social desirability biases, potentially impacting data reliability and overall study conclusions. These issues were mitigated by using validated self-reporting tools (RMQ, DPQ, and VAS).

The 6-week follow-up period was sufficient to observe several impacts on LBP symptoms in clinically relevant ranges; however, it may be too short to fully capture all therapeutic benefits and to assess the stability of these benefits over time. For instance, nonspecific chronic neck pain has shown improvement with combined manual therapy and physical exercise over treatment periods ranging from 6 weeks to 3 months, as demonstrated in a recent randomized controlled trial.^[38]^ Additionally, the residual pain intensity observed after the second session (19.7 ± 21.3 mm) suggests that additional sessions may yield further improvement.

Due to the observational and non-interventional study design, treatment application was left to the practitioner’s discretion, potentially introducing variability in treatment application and possible inconsistencies in outcomes. However, to participate, the care program was limited to 2 therapy sessions to ensure a minimum of homogeneity in participant management.

Patients receiving additional sessions or complementary therapies were not included. This raises the question of how this therapy might perform within a comprehensive care plan that includes other approaches not explored in this study. Therefore, it is challenging to generalize these findings to all care protocols for nonspecific LBP, including those incorporating manual therapy and acupressure.

### 4.3. Interpretation

A strength of our study is its focus on participants experiencing LBP despite 4 weeks of physical exercise and who had not yet progressed to the chronic stage. At study inclusion, the median LBP duration was 12 weeks, exceeding the usual 4-week period sufficient to resolve most nonspecific LBP,^[39]^ and a point at which 4% to 25% of LBP cases may become chronic (three months post-diagnosis).^[40]^

Manual therapy has shown therapeutic interest primarily in chronic LBP,^[13–15]^ and acupressure has been used to relieve pain,^[16,18]^ notably labor pain^[41]^ and LBP.^[17]^ Recent studies highlight the efficacy of various LBP treatments. Kasimis et al^[14]^ demonstrated that manual therapy alone reduced pain by up to 35.2% and improved functional disability by 36.4% 1 month posttreatment. Similarly, Yeh et al^[17]^ reported that auricular point acupressure, administered over 4 sessions within 28 days, resulted in a significant average pain reduction of 58.5%. Schmidt et al^[13]^ found that combining manual treatment for trigger points with kinesio taping significantly alleviated pain by 42.0% 1 month after a 3-week treatment for pelvic obliquity. Er et al^[15]^ implemented a comprehensive 4-week protocol with hot packs, exercise, and connective tissue massage, achieving pain reductions of 84.6% and 93.0% for connective tissue massage and classical massage, respectively. In our study, we observed a pain reduction of 49.0% and a functional disability improvement of 51.9% by Day 21, an effect size consistent with previous studies. This supports that the observed pain reduction in our study may not be solely attributable to a time or Hawthorne effect but could be, at least partially, a result of the treatment’s potential effectiveness. The reduction of lumbar pain to a clinically relevant threshold^[36,37]^ and the improvement in functional disability suggest that this method, combining manual therapy with acupressure, could serve as a therapeutic option to prevent chronic LBP onset and its associated disabling consequences.

We also considered chronicity risk at study inclusion and conducted an exploratory analysis by stratifying participants into 3 subgroups. This analysis indicated that all participants benefited from the therapy, with symptom improvements progressing significantly, irrespective of the baseline chronicity risk level. This finding suggests that chronic LBP risk factors (e.g., anxiety, prolonged posture, weight issues) should not prevent the prescription of manual therapy and acupressure. Additionally, this method may indirectly affect factors exacerbating LBP chronicity, such as anxiety, as indicated by the significant decrease in the impact of pain on anxiety-depression.

Another strength of this study is the inclusion of a substantial number of participants (N = 114), exceeding that of recent observational^[42]^ or prospective studies^[13–15]^ evaluating manual therapy and acupuncture for LBP. This sample size allowed us to reasonably capture the inter-individual variability inherent to the target population.

### 4.4. Generalizability

Being observational, non-interventional, and lacking a control group, the generalizability of this study’s findings is limited. The discretion left to practitioners regarding treatment modalities introduced variability in the intervention’s application, potentially leading to inconsistencies in outcomes and challenges in replicating or generalizing the results. However, the study provides valuable insights into potential treatment effectiveness. One unique contribution of this study is its integration of manual therapy and acupressure techniques, offering real-life data supporting their combined efficacy for treating nonspecific LBP. The interdisciplinary approach of the Biokinergie® method enhances understanding in complementary medicine and encourages interdisciplinary strategies in pain management. These preliminary findings lay the groundwork for further experimental research. The next step should involve randomized controlled trials incorporating blinding, an extended follow-up period, and an assessment of long-term effects to establish more conclusive evidence.

## 5. Conclusion

After 2 sessions combining manual therapy and acupressure, participants with nonspecific LBP experienced a statistically and clinically significant reduction in lumbar pain intensity, along with improvements in functional disability and QoL. Participants expressed high satisfaction with their care and management. However, further experimental studies on the Biokinergie® method are warranted to confirm its efficacy in pain relief. Large-scale randomized controlled trials should consider methodological design aspects, including treatment duration, placebo effects, and the integration of this approach into a comprehensive care plan.

## Acknowledgments

We would like to thank all the patients who agreed to participate in this study. We also thank all the investigators for their contributions. We thank Camille DEJOS for drafting the article, Xavier SIOMBOING for the statistical analyses, and the team at Soladis Clinical Studies for their support in conducting the study.

## Author contributions

**Conceptualization:** Gilles Ducret, Yann Fardini, Sandrine Vejux.

**Funding acquisition:** Gilles Ducret.

**Investigation:** Marc Guillaume, Sandrine Vejux, Hassène Chaabi.

**Methodology:** Marc Guillaume, Yann Fardini, Sandrine Vejux.

**Project administration:** Gilles Ducret, Marc Guillaume, Sandrine Vejux, Hassène Chaabi.

**Resources:** Gilles Ducret, Marc Guillaume, Sandrine Vejux, Hassène Chaabi.

**Supervision:** Gilles Ducret, Marc Guillaume, Sandrine Vejux, Hassène Chaabi.

**Validation:** Gilles Ducret, Marc Guillaume, Yann Fardini, Sandrine Vejux, Hassène Chaabi.

**Visualization:** Yann Fardini.

**Writing – original draft:** Yann Fardini.

**Writing – review & editing:** Gilles Ducret, Marc Guillaume, Sandrine Vejux, Hassène Chaabi.

## Supplementary Material


