# Supplementary material for: Assessment of a manual therapy and acupressure method as a treatment of nonspecific low back pain: A prospective, observational and non-interventional cohort study
Source: Medicine (Baltimore). 2024 Dec 20;103(51):e40891. doi: 10.1097/MD.0000000000040891 (PMC11666163; doi:10.1097/MD.0000000000040891)
Supplement: Supplementary file 1 [file medi-103-e40891-s001.docx]

**Supplementary Table S1. Secondary outcomes - Participants’ satisfaction with care delivery and with treatment outcome expectations**

|  | | **Full-Analysis Set N = 114** |
| --- | --- | --- |
| **Participants satisfaction with care delivery** | | |
| Day 0 | N (missing) | 108 (6) |
| -Very satisfied | n (%) | 84 (77.8%) |
| -Satisfied | n (%) | 18 (16.7%) |
| -Neutral | n (%) | 1 (0.9%) |
| -Dissatisfied | n (%) | 1 (0.9%) |
| -Very dissatisfied | n (%) | 4 (3.7%) |
| Day 21 | N (missing) | 108 (6) |
| -Very satisfied | n (%) | 90 (83.3%) |
| -Satisfied | n (%) | 13 (12.0%) |
| -Neutral | n (%) | 2 (1.9%) |
| -Dissatisfied | n (%) | 0 (0.0%) |
| -Very dissatisfied | n (%) | 3 (2.8%) |
|  |  |  |
| **Participants satisfaction with treatment outcome expectations** |  |  |
| Day 42 | N (missing) | 106 (8) |
| -Very satisfied | n (%) | 60 (56.6%) |
| -Satisfied | n (%) | 29 (27.4%) |
| -Neutral | n (%) | 12 (11.3%) |
| -Dissatisfied | n (%) | 2 (1.9%) |
| -Very dissatisfied | n (%) | 3 (2.8%) |

**Supplementary Table S2. Exploratory outcomes - Evolution of pain, functional disability, and impact of low back pain on the participants’ quality of life, as a function of the risk of LBP chronicity**

|  | | **Risk of chronicity** | | |
| --- | --- | --- | --- | --- |
|  | | **Low N = 20** | **Intermediate N = 38** | **High N = 54** |
| **Mean intensity of pain over the last 24 hours** | |  |  |  |
| Day 0 | n (missing) | 20 (0) | 38 (0) | 54 (0) |
|  | Mean (± SD) | 47.5 (±17.2) | 59.8 (±12.6) | 67.0 (±16.8) |
| Day 24 | n (missing) | 20 (0) | 36 (2) | 51 (3) |
|  | Mean (± SD) | 25.3 (±18.8) | 22.4 (±17.2) | 25.7 (±26.4) |
| Difference Day 24-Day 0 | n (missing) | 20 (0) | 36 (2) | 51 (3) |
|  | Mean (± SD) | -22.3 (±24.7) | -37.8 (±20.7) | -40.7 (±30.7) |
| MMRM: Pain change from baseline (Day 24-Day 0) | Estimation (± SEM) | -32.0 (±4.8) | -23.6 (±3.4) | -25.1 (±2.9) |
|  | 95% CI | [-41.5;-22.5] | [-30.3;-16.9] | [-30.9;-19.3] |
| Day 42 | n (missing) | 20 (0) | 34 (4) | 51 (3) |
|  | Mean (± SD) | 22.2 (±20.0) | 20.5 (±18.9) | 18.4 (±23.8) |
| Difference Day 42-Day 0 | n (missing) | 20 (0) | 34 (4) | 51 (3) |
|  | Mean (± SD) | -25.4 (±29.0) | -38.8 (±23.0) | -48.0 (±29.3) |
| MMRM: Pain change from baseline (Day 42-Day 0) | Estimation (± SEM) | -37.6 (±5.1) | -39.7 (±3.7) | -42.8 (±3.1) |
|  | 95% CI | [-47.7;-27.4] | [-47.0;-32.4] | [-49.0;-36.6] |
| Statistical test – Fixed effect of the time | | *P* < .001 | | |
| Statistical test – Fixed effect of risk of chronicity score | | *P* = .82 | | |
| Statistical test – Fixed effect of the interaction time x risk of chronicity score | | *P* = .18 | | |
| **Functional disability** |  |  |  |  |
| Day 0 | n (missing) | 20 (0) | 38 (0) | 54 (0) |
|  | Mean (± SD) | 6.3 (±5.0) | 9.3 (±4.2) | 13.3 (±4.5) |
| Day 42 | n (missing) | 20 (0) | 35 (3) | 51 (3) |
|  | Mean (± SD) | 2.5 (±2.9) | 2.9 (±3.0) | 3.7 (±4.7) |
| Difference Day 42-Day 0 | n (missing) | 20 (0) | 35 (3) | 51 (3) |
|  | Mean (± SD) | -3.9 (±4.5) | -6.1 (±4.8) | -9.6 (±6.1) |
| *MMRM:* Functional disability change from baseline (Day 42-Day 0) | Estimation (± SEM) | -6.3 (±1.0) | -5.8 (±0.7) | -4.8 (±0.6) |
|  | 95% CI | [-8.4; -4.3] | [-7.2; -4.4] | [-6.1; -3.5] |
| Statistical test – Fixed effect of the time | | *P* < .001 | | |
| Statistical test – Fixed effect of risk of chronicity score | | *P* = .82 | | |
| Statistical test – Fixed effect of the interaction time x risk of chronicity score | | *P* = .05 | | |
| **Daily activities** |  |  |  |  |
| Day 0 | n (missing) | 20 (0) | 38 (0) | 54 (0) |
|  | Mean (± SD) | 39.0 (±17.5) | 54.8 (±16.5) | 63.2 (±16.6) |
| Day 42 | n (missing) | 20 (0) | 35 (3) | 50 (4) |
|  | Mean (± SD) | 14.3 (±14.5) | 22.20 (±21.3) | 19.7 (±24.5) |
| Difference Day 42-Day 0 | n (missing) | 20 (0) | 35 (3) | 50 (4) |
|  | Mean (± SD) | -24.8 (±22.2) | -32.6 (±23.9) | -42.8 (±30.8) |
| MMRM: LBP impact change from baseline (Day 42-Day 0) | Estimation (± SEM) | -33.5 (±5.2) | -27.1 (±3.5) | -25.1 (±3.1) |
|  | 95% CI | [-43.8; -23.2] | [-34.0; -20.1] | [-31.3; -19.0] |
| Statistical test – Fixed effect of the time | | *P* < .001 | | |
| Statistical test – Fixed effect of risk of chronicity score | | *P* = .59 | | |
| Statistical test – Fixed effect of the interaction time x risk of chronicity score | | *P* = .16 | | |
| **Work-leisure activities** |  |  |  |  |
| Day 0 | n (missing) | 20 (0) | 38 (0) | 54 (0) |
|  | Mean (± SD) | 28.5 (±25.6) | 48.6 (±22.5) | 61.7 (±25.8) |
| Day 42 | n (missing) | 20 (0) | 35 (3) | 49 (5) |
|  | Mean (± SD) | 12.8 (±16.2) | 21.1 (±22.5) | 19.3 (±26.4) |
| Difference Day 42-Day 0 | n (missing) | 20 (0) | 35 (3) | 49 (5) |
|  | Mean (± SD) | -15.8 (±27.5) | -27.3 (±27.6) | -41.7 (±31.5) |
| *MMRM:* LBP impact change from baseline (Day 42-Day 0) | Estimation (± SEM) | -27.8 (±5.4) | -28.5 (±3.7) | -24.2 (±3.3) |
|  | 95% CI | [-38.5; -17.0] | [-35.9; -21.1] | [-30.7; -17.7] |
| Statistical test – Fixed effect of the time | | *P* = .03 | | |
| Statistical test – Fixed effect of risk of chronicity score | | *P* = .99 | | |
| Statistical test – Fixed effect of the interaction time x risk of chronicity score | | *P* = .11 | | |
| **Anxiety-depression** |  |  |  |  |
| Day 0 | n (missing) | 20 (0) | 38 (0) | 54 (0) |
|  | Mean (± SD) | 20.3 (±25.0) | 31.8 (±23.4) | 40.4 (±26.9) |
| Day 42 | n (missing) | 20 (0) | 35 (3) | 49 (5) |
|  | Mean (± SD) | 8.5 (±15.7) | 12.3 (±18.5) | 10.1 (±17.5) |
| Difference Day 42-Day 0 | n (missing) | 20 (0) | 35 (3) | 49 (5) |
|  | Mean (± SD) | -11.8 (±23.4) | -20.9 (±21.4) | -28.7 (±29.5) |
| *MMRM:* LBP impact change from baseline (Day 42-Day 0) | Estimation (± SEM) | -18.5 (±3.9) | -16.1 (±2.8) | -17.2 (±2.4) |
|  | 95% CI | [-26.2; -10.8] | [-21.6; -10.6] | [-21.9; -12.4] |
| Statistical test – Fixed effect of the time | | *P* = .004 | | |
| Statistical test – Fixed effect of risk of chronicity score | | *P* = .70 | | |
| Statistical test – Fixed effect of the interaction time x risk of chronicity score | | *P* = .31 | | |
| **Social life** |  |  |  |  |
| Day 0 | n (missing) | 20 (0) | 38 (0) | 54 (0) |
|  | Mean (± SD) | 10.5 (±14.9) | 17.4 (±18.6) | 26.9 (±24.3) |
| Day 42 | n (missing) | 20 (0) | 35 (3) | 49 (5) |
|  | Mean (± SD) | 2.3 (±6.0) | 6.1 (±12.1) | 7.2 (±15.5) |
| Difference Day 42-Day 0 | n (missing) | 20 (0) | 35 (3) | 49 (5) |
|  | Mean (± SD) | -8.3 (±12.8) | -12.4 (±17.8) | -17.3 (±24.2) |
| MMRM: LBP impact change from baseline (Day 42-Day 0) | Estimation (± SEM) | -10.13 (±2.9) | -9.5 (±2.1) | -10.0 (±1.8) |
|  | 95% CI | [-15.8; -4.4] | [-13.6; -5.4] | [-13.6; -6.5] |
| Statistical test – Fixed effect of the time | | *P* = .003 | | |
| Statistical test – Fixed effect of risk of chronicity score | | *P* = .93 | | |
| Statistical test – Fixed effect of the interaction time x risk of chronicity score | | *P* = .97 | | |
